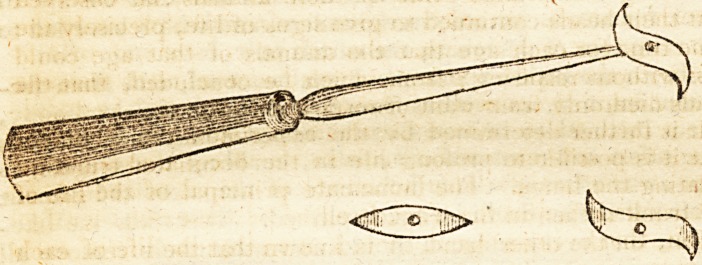# Mr. Jukes's Observations on Removing Cartilage

**Published:** 1812-05

**Authors:** Alfred Jukes

**Affiliations:** London


### *To the Editors of the Medical and Physical Journal.*

(With an Engraving.)

GENTLEMEN,

SURGEONS have long differed in opinion respecting the propriety of removing the cartilaginous surface, necessarily exposed in amputations at the various joints of the human body; and, how far it may prove advantageous or not, is still a question of doubt even with the most eminent of the profession.

From such diversity of opinion, I may augur that the result from suffering the cartilage to remain, has been very various as to success; and, under this impression, I have been instigated to trouble you with a few remarks on this subject.

I shall, however, confine my observations to amputations of the arm at the shoulder-joints, as demanding more particular attention than those of the hip-joint; the former being of frequent, the latter of very rare, occurrence.

In entering upon this inquiry, an important distinction of cases requiring this operation strikes me as being highly proper to be made, as respects the absolute necessity of the removal of the cartilage, viz. cases where it becomes neces-

---

sary sary to operate from accidental injuries, from those produced by the effects of long pre-existing disease. In the former it probably takes place in an healthy body, and the joint in a perfectly sound state. Under such circumstances, together with the frequent occasion for rapidity of operating, they are all favorable occurrences towards effecting a speedy union of the parts, even where the cartilage is suffered to remain; and cases corroborating its frequent success are not scanty.

Among those, however, which most frequently occur in private practice, the major part of them are referable to the last division of cases; and it is amidst these that we are to look for any untoward circumstances, and which have led to an adoption of detaching the cartilage.

From the little experience I can boast of, and from frequent inquiry, as well as from operating on the dead body, I am inclined to believe that the removal of the cartilage is the safest and most judicious plan, enabling the surgeon to cut away more of the attachments of the capsular ligament than otherwise could be accomplished. It strikes me likewise, that every surgeon should very attentively remove all remaining portions of synovial membrane that are within his reach; these acting, probably, in a much greater degree unfavorably than the cartilage itself.

In all diseases of joints commonly arising from a scrofulous habit, a morbid secretion of synovia always takes place; and, when we reflect on the numerous bursæ mucosæ which are situated around, and connected with, this joint, through the medium of the capsular ligament, the necessity of a clean removal becomes apparent. And, though the parts will sometimes heal without these precautions, yet subsequent tumefactions from a redundancy of synovia are oftentimes the consequence, requiring an outlet for its discharge, until such time as the morbid secretion shall subside. These, together with the frequent separation of the cartilage, rather than its disappearance by absorption, are sufficient reasons to prove, that in many instances the removal of it is very judicious practice.

From the foregoing remarks, it appears to me of considerable importance that some defined rule of conduct in such cases should be adopted; since it is very probable surgeons, wavering between the two prevailing opinions, have taken the medium (and which I have seen practised), by merely abrading the surface of the cartilage with the knife; tending, perhaps, to the prejudice rather than to the benefit of the patient, by producing subsequent separation.

I trust I shall not be considered presumptuous in stating, that that I would myself, under all circumstances, remove the cartilage; such a precaution, in my estimation, being most likely a prevention of inconvenience which might otherwise arise. Having, on many occasions, experienced much trouble and delay in separating the cartilage by the common scalpel, I was led to devise an instrument (the design of which, accompanying these remarks, I offer for your consideration,) seemingly far better calculated for separating cartilage at this particular joint; and its being the first instrument adapted particularly to this purpose, it probably may not prove unworthy of your notice.

It consists of a perforator, the extremity of which, being a screw, admits of the application of the blade, the cutting edges of which are reversed as to each other;. and, being curved, is an advantage so far as to render it less liable to be broken, the point being more gradually acted upon than if straight. In its application upon the dead body, it appears to me to have the advantage of detaching the remaining portions of the capsular ligament, and to cut away the centre of it, a circumstance I could never accomplish with the scalpel. The blades may be made of various shapes and dimensions, and applied as occasion may require. If, upon perusal, you should consider this worthy its insertion in your Journal, you will greatly oblige,

Your's, respectfully,

*London, March* 30, 1812. ALFRED JUKES.

## Figures and Tables

**Figure f1:**